# Differential contributions of ApoE4 and female sex to BACE1 activity and expression mediate A**β** deposition and learning and memory in mouse models of Alzheimer’s disease

**DOI:** 10.3389/fnagi.2015.00207

**Published:** 2015-10-31

**Authors:** Xu Hou, Samuel O. Adeosun, Qinli Zhang, Brett Barlow, Melissa Brents, Baoying Zheng, Junming Wang

**Affiliations:** ^1^Program in Neuroscience, University of Mississippi Medical Center, JacksonMS, USA; ^2^Department of Pathology, University of Mississippi Medical Center, JacksonMS, USA; ^3^Department of Psychiatry and Human Behavior, University of Mississippi Medical Center, JacksonMS, USA; ^4^Center of Memory Impairment and Neurodegenerative Dementia, University of Mississippi Medical Center, JacksonMS, USA

**Keywords:** sex-dependent effect, ApoE4, BACE1, learning and memory, Alzheimer’s Disease

## Abstract

Alzheimer’s disease (AD), the most common form of dementia, disproportionately affects women in both prevalence and severity. This increased vulnerability to AD in women is strongly associated with age-related ovarian hormone loss and apolipoprotein E 4 allele (ApoE4), the most important genetic risk factor for sporadic AD. Up to date, the mechanism involved in the interaction between ApoE4 and sex/gender in AD is still unclear. This study evaluated the sex-dependent ApoE4 effects on learning and memory, Aβ deposition and potential mechanisms, using mice bearing both sporadic (ApoE4) and familial (APP_Swe_, PS1_M146V_, tau_P301L_; 3xTg) AD risk factors and compared with sex- and age-matched 3xTg or nonTg mice. Compared to nonTg mice, transgenic mice of both sexes showed spatial learning and memory deficits in the radial arm water maze and novel arm discrimination tests at 20 months of age. However, at 10 months, only ApoE4/3xTg mice showed significant learning and memory impairment. Moreover, molecular studies of hippocampal tissue revealed significantly higher protein levels of Aβ species, β-site APP cleavage enzyme (BACE1) and Sp1, a transcription factor of BACE1, in female ApoE4/3xTg when compared with female nonTg, female 3xTg, and male ApoE4/3xTg mice. Significantly increased BACE1 enzymatic activities were observed in both male and female mice carrying ApoE4; however, only the females showed significant higher BACE1 expressions. Together, these data suggest that ApoE4 allele is associated with increased BACE1 enzymatic activity, while female sex plays an important role in increasing BACE1 expression. The combination of both provides a molecular basis for high Aβ pathology and the resultant hippocampus-dependent learning and memory deficits in female ApoE4 carriers.

## Introduction

Alzheimer’s disease, a devastating neurodegenerative disorder, accounts for 60–80% of all dementia among individuals who are ≥65 years of age (Barker et al., 2002; Blennow et al., 2006; Wilson et al., 2012). The magnitude of its reach is shown both through its standing as the third leading cause of death in the United States (US) and through the immense burden of care associated with its progression (Hurd et al., 2013; Freedman and Spillman, 2014; James et al., 2014). Moreover, among the top ten causes of death reported by the US National Center for Health Statistics (2015) AD remains the only one that cannot be prevented or cured. Furthermore, AD incidence rate is increasing, and it is expected that by 2050, 13.8 million of the US population will be living with AD (Hebert et al., 2013).

Multifactorial etiologies of AD link it to multiple risk factors rather than a single cause. Epidemiological studies have shown gender differences in both the incidence and severity of AD, with women at much greater risk, even after adjusting for longer life span and education levels (Fratiglioni et al., 1997; Jorm and Jolley, 1998; Andersen et al., 1999; Fratiglioni et al., 2000; Barker et al., 2002). Compared to affected men, women with AD have significant increases in global neuropathology and worse dementia ratings (Barnes et al., 2005). Meanwhile, the ɛ4 allele of the ApoE has been strongly confirmed as a genetic risk factor for sporadic AD (Raber et al., 2004; Loy et al., 2014), with the other two isoforms, ɛ2 and ɛ3, respectively, decreasing the likelihood of AD or having a neutral effect. ApoE4 carriers are at a 3- to 12-fold higher risk of developing AD than ApoE3 carriers, depending on gene dose (Corder et al., 1993). ApoE4 carriers also experience earlier onset, faster progression, and more pronounced deficits during disease development than ApoE3 carriers (Raber et al., 2004; Cosentino et al., 2008; Sando et al., 2008; Reinvang et al., 2013). Moreover, ApoE4 and gender have prominent interaction, with the ApoE4 link to AD far more pronounced in women than in men (Corder et al., 2004; Fleisher et al., 2005; Lehmann et al., 2006; Damoiseaux et al., 2012; Altmann et al., 2014; Ungar et al., 2014). Women with ApoE4 experience greater gross hippocampal pathology, more severe memory deficit, lower cognitive scores, lower hippocampal volumes, more senile plaque, and higher spinal fluid levels of tau, along with a higher risk for AD than men (Bretsky et al., 1999; Corder et al., 2004; Fleisher et al., 2005; Lehmann et al., 2006; Damoiseaux et al., 2012; Altmann et al., 2014). Similar sex differences are also shown in transgenic mouse models of AD, with females showing earlier and more robust deficits than males. For example, older female mice with familiar AD (FAD) mutations exhibit higher brain amyloid β (Aβ) levels and poorer cognitive performance than age-matched males (Wang et al., 2003; Halford and Russell, 2009; Carroll et al., 2010). Age-dependent ApoE4-induced impairments in spatial learning and memory are also seen in female, but not in male mice (Raber et al., 1998; Raber et al., 2000; Bour et al., 2008; Reverte et al., 2012). Our search of the literature, however, yielded few cognitive assessments of ApoE4-sex effects in transgenic mice with FAD mutations. Such studies are needed, as these mutations have been shown to produce similar AD pathology seen in human brains (Johnson-Wood et al., 1997; Oddo et al., 2003; LaFerla and Green, 2012).

Although the sex-dependent ApoE4 effects on cognitive function are strongly associated with increased Aβ levels in AD brains (Corder et al., 2004; Liu et al., 2014; Ungar et al., 2014), the underlying molecular mechanisms are still unclear. Excessive Aβ accumulation, one of the neuropathological hallmarks in AD, is produced through amyloidogenic pathway via a sequential cleavage of the APP by β-secretase and γ-secretase (Selkoe, 2001; Vassar, 2004). Evidence from animal and cell culture studies suggests that the β-site APP cleavage enzyme (BACE1) is the major β-secretase in the brain, serving as the rate-limiting enzyme in amyloidogenesis (Cai et al., 2001; Luo et al., 2001; Li et al., 2006; McConlogue et al., 2007; Cole and Vassar, 2008; Cai et al., 2012). Increased BACE1 levels and activities have been shown in AD brains (Fukumoto et al., 2002; Yang et al., 2003; Li et al., 2004), with a further increase in cerebrospinal fluid (CSF) or brains of AD subjects with ApoE4 (Stockley et al., 2006; Ewers et al., 2008). However, other studies showed no difference in BACE1 activity or reduced BACE1 levels in ApoE4 carriers (Mulder et al., 2010; Decourt et al., 2013). Nevertheless, few of these studies have included sex differences as confounding factors.

To investigate how the interaction of ApoE4 and sex contributes to cognitive impairment and to BACE1-dependent amyloidogenesis, as well as to identify possible mechanisms underlying this interaction, we compared behavioral and molecular measures in an AD mouse model carrying the human ApoE4 allele plus triple transgenes with FAD mutations (PS1_M146V_, APP_Swe_, and tau_P301L_; 3xTg). These familial mutations are known to produce both Aβ plaques and neurofibrillary tangles in the aging mouse brain (Oddo et al., 2003). To distinguish possible contributions from ApoE4, all results were compared with 3xTg and nonTg background controls. Our results suggest that the combination of ApoE4 allele and female sex contributes to the increased BACE1 enzymatic activity and expression, respectively. It provides a molecular basis for high Aβ pathology and the resultant hippocampus-dependent learning and memory deficits in female ApoE4 carriers.

## Materials and Methods

### Animal

All procedures were in compliance with the University of Mississippi Medical Center Institutional guidelines, approved by the UMMC Institutional Animal Care and Use Committee (IACUC) and in accordance with NIH guidelines for the use of vertebrate animals.

A total of 156 mice used in this study were age (10 and 20 month-old) and sex matched nonTg, 3xTg (PS1_M146V_, APP_Swe_, and tau_P301L_) and ApoE4/3xTg (PS1_M146V_, APP_Swe_, tau_P301L_, and hApoE4) mice. The mice were obtained from the laboratory of Dr. Frank LaFerla of the University of California, Irvine. The animals were maintained at the University of Mississippi Medical Center, group housed in a temperature/humidity controlled environment with free access to food and water, and kept on a 12 h light/dark cycle with lights on at 6 a.m. and lights off at 6 p.m. The number of animals used in different studies are listed in **Table 1**.

**Table 1:** Experimental design.

Study	Genotype	Sex	Age	
Age and genotype effects in AD mice	nonTg	Male and female	10 M (*n* = 9)	20 M (*n* = 8)
	3xTg	Male and female	10 M (*n* = 10)	20 M (*n* = 8)
	ApoE4/3xTg	Male and female	10 M (*n* = 8)	20 M (*n* = 8)
Sex and age effects in ApoE4/3xTg mice	ApoE4/3xTg	Male	10 M (*n* = 10)	20 M (*n* = 9)
		Female	10 M (*n* = 10)	20 M (*n* = 8)
Sex and genotype effects in AD mice	nonTg	Male	10 M (*n* = 12)	
		Female	10 M (*n* = 10)	
	3xTg	Male	10 M (*n* = 12)	
		Female	10 M (*n* = 12)	
	4xTg	Male	10 M (*n* = 12)	
		Female	10 M (*n* = 10)	

*The number of animals used in studies for age-, sex- and genotype-dependent differences in AD mice.*

3xTg mice were generated by microinjecting two independent transgene constructs that encode human APP_Swe_ and tau_P301L_ into single-cell embryos, and then harvested from mutant homozygous PS1_M146V_ knockin mice (Oddo et al., 2003). To determine the effect of replacing mouse ApoE with human ApoE4, 3xTg mice were crossed with the human ApoE4 knockin mice to generate ApoE4/3xTg mice (Oddo et al., 2009), in which the human ApoE4 gene is expressed under the control of murine ApoE promotor (Knouff et al., 1999).

### Vaginal Smear

The estrus cycle stage and fertility of the female mice, across all age groups, were determined by collecting samples of vaginal mucosa at the same time (10 am) each day for 2 weeks, as described by McLean et al. (2012). Each vaginal mucosa sample was slightly smeared on a single glass slide, allowed to dry completely at room temperature, and then subjected to crystal violet staining by immersion in crystal violet for 1 min. This process was followed by destaining in ddH_2_O for 1 min, after which the stained smear was topped with glycerol, covered by a coverslip, and observed under a light microscope. Phases of the estrus cycle (proestrus, estrus, metestrus, diestrus) were determined based on the mucosal cell types identified in the samples.

### Radial Arm Water Maze

The RAWM, a behavioral test described by Alamed et al. (2006) for evaluating learning and spatial/working memory, was slightly modified, as previously described (Adeosun et al., 2013). Our apparatus consisted of a 1m diameter pool, divided into five alleys (Supplementary Figure S1), each a navigable alley of 40 cm length and 15–20 cm width leading to a 22 cm diameter circle at the pool’s center. In one arm (the target arm), a 10 cm × 10 cm square transparent platform was placed 5 cm from the wall and 0.5 cm beneath the water’s surface to serve as the target throughout all training trials.

During training, each mouse was gently placed into one of the four arms (but *not* the target arm) and allowed a maximum time of 60 s to find the platform hidden in the target arm; if the platform was not located during this time, we gently guided the mouse to it. Although the platform’s location was altered for different animals, it was consistently maintained for each individual animal until the end of the training session. During 2 days of RAWM testing, working with cohorts of 4–5 mice at a time, a total of eight training blocks were performed, with two training trials per animal per block, for a total of 16 attempts by each animal. The start arm was randomized throughout the eight training blocks. In all cases, the numbers of wrong arm entries (errors), along with time to find the platform (latency), were recorded. One hour after the end of the training, the hidden platform was removed and a 1 min probe trial was performed. The probe trial was recorded, and the video was analyzed using Noldus EthoVision XT 8.5 software (Noldus, Leesburg, VA, USA). Data from animals that remain in the start arm for the entire probe trial were excluded from the results. In the RAWM, the average latency and error from each block was compared to the corresponding data from the first training block and also compared between each genotype using two-way ANOVA and *post hoc* test. The percentage of time that each animal spent in the target arm (T) was calculated as follows: T% = (100% xT)/(time spent in all the alleys). T% was compared to a 20% chance level using a one-sample T-test to show whether or not the animal was able to learn and remember the location of the hidden platform, and was also compared using two-way ANOVA to examine the sex and genotype effects among groups.

### Novel Arm Discrimination

To study spatial memory, which is hippocampus-dependent, we used the NAD task (Place memory task) (Supplementary Figure S2). This test is based on the natural tendency of rodents to show preference for a novel environment over a familiar one. As previously described (Adeosun et al., 2013), the test was carried out in the Y-maze apparatus, using a floor covered with the wood chippings used in mice cages. Different objects were placed to serve as extra-maze cues at various distances around the maze. The test involved two trials: an acquisition and a retention trial, separated by a 1 h ITI. During acquisition trials, one arm was blocked. The blocked arm was the Novel arm (N) and the other arm was the Familiar arm (F); the remaining arm was the Start arm (S), which remained open and was kept constant in both trials. In the acquisition trial, each animal was placed in the Start arm of the maze and then allowed to explore both the Start and the Familiar arms (F) for 5 min. After 1 h, the Novel arm (N) was opened and the animal was allowed to explore again for 5 min. The walls of the maze were cleaned with 70% alcohol and the wood chippings covering the floor of the maze were mixed before the next animal was placed in the maze. While the Start arm (S) was kept constant for all animals, the Novel and Familiar arms were counterbalanced among the animals within each group. The experiment was recorded with the Noldus system and the video was analyzed oﬄine using Noldus EthoVision XT 8.5 software. Data from animals that remain in the start arm for either acquisition or retention trial were excluded from the results. In the NAD, the percentage of time spent in N was calculated as follows: N% = (100% × N)/(N+F). N% was compared to a 50% chance level using a one-sample *t*-test. Whether or not the animal discriminated between the novel and other arm was indicted by an inequality in the time spent in either arm. N% was also compared using two-way ANOVA to determine the sex and genotype effects between groups.

### Tissue Collection

Before euthanasia, mice were anesthetized with isoflurane. After vascular flushing by cardiac perfusion using saline, brains were harvested on an ice-cooled plate. The hippocampus was dissected from the right hemispheres and immediately wrapped in pre-labeled aluminum foils, snap-frozen in liquid nitrogen, and stored at -80°C until ready for protein extraction.

In each experimental group, three hippocampal samples were subjected to the extraction buffer for β-secretase activity assay, and rest of the samples were subjected to RIPA buffer for protein extraction. The statistical significance of the following molecular studies was assessed by two-way ANOVA and a subsequent Bonferroni *post hoc* test. Differences were considered significant at probability (P) values less than 0.05.

### Protein Extraction

Protein was extracted from the hippocampus sample as previously described (Adeosun et al., 2012), with modifications. Briefly, each samples was homogenized in 200 μl of cold DEPC water with a pestle motor mixer (MIDSCI, St. Louis, MO, USA) for 20 s. A 135 μl of the homogenate was immediately transferred into a tube already containing 135 μl of RIPA-HALT protease inhibitor cocktail mix (1:100) (Halt from Thermo-scientific; Rockford, IL, USA). The homogenate-RIPA mix was left at 4°C for 30 min and centrifuged at 11,000 rpm at 4°C in a refrigerated centrifuge (Eppendorf, Hauppauge, NY, USA) for 12 min. The supernatant was then transferred into a fresh tube and centrifuged again at 11,000 rpm for 15 min at 4°C. This supernatant was then again transferred into the final protein tube as cytoplasmic protein. The pellet from the first centrifugation was first washed twice with PBS and then sonicated (Qsonica, LLC, Newtown, CT, USA) three times with an additional 50 μl of RIPA-HALT at 15A for 10 s to release nuclear protein. The protein concentration of each sample was determined by the BCA Protein Assay Kit (Pierce Biotechnology, Rockford, IL, USA) before being prepared for gel electrophoresis.

### SDS PAGE and Immunoblotting

Protein extracts were prepared using RIPA-Halt mix to attain the same final concentration for gel electrophoresis. Three parts of protein were then mixed with one part 4× laemmli buffer (BP-110R, Boston BioProducts, Ashland, MA, USA). The protein-laemmli mix was heated at 95°C, while mixing, for 5 min; it was then allowed to cool on ice for 2 min. A Mini-PROTEAN Tetra cell (Bio-Rad, Hercules, CA, USA) was used as a running chamber for vertical gel electrophoresis. Cytoplasmic proteins of six samples per group were used for Aβ and BACE1 detection, and nuclear proteins were used for Sp1 detection. Samples (30 μg/well) were loaded on a 10% polyacrylamide gel, along with the precision protein plus western blot ladder (Bio-rad, Hercules, CA, USA), and run at 90 V for 2 h. The protein was transferred to a PVDF membrane using the Trans-Blot Turbo Transfer System (Bio-rad, Hercules, CA, USA). Next, the protein was blotted on a PVDF membrane with Pierce Fast Western Blot Kit, ECL Substrate (Pierce Biotechnology, Rockfod, IL, USA), which contains washing buffer, antibody diluent, optimized HRP reagent, and ECL Detection Reagents. Specifically, the membrane was washed with washing buffer and incubated with primary antibody BACE1 (D10E5, 5606S, 1:250, Cell Signaling) and Sp1 (1C6, sc-420, 1:100, Santa Cruz) for 30 min at room temperature. The membrane was then immediately incubated with the optimized HRP reagent at 1:10 dilution for 10 min. After washing three times, the membrane was incubated with ECL substrate for 5 min and the chemiluminiscent signal detected by the Bio-Rad ChemiDoc^TM^ XRS+ system (Bio-Rad, Hercules, CA, USA) with Image lab 3.0 software every 30 s for 10 min. Protein sample loading was normalized with β-Actin (AC-15, ab6276, 1:10000, Abcam), detected on the same membrane after stripping. Data were presented as relative optical densities of the individual bands ± SEM. In addition to Two-way ANOVA and *post hoc* test, a correlation between Sp1 and BACE1 protein expression was also examined.

### Dot Blot

Dot blot was used to detect the protein expression of Aβ species in the hippocampal tissues. Proteins extracts of four samples per group were normalized with RIPA-Halt mix to reach the same final concentration and used in the dot blot. Specifically, the grid was drawn on a pre-cut nitrocellulose membrane by pencil to indicate the region for each sample to blot. Using narrow-mouth pipette tip, 30 μg of samples were spotted onto the membrane at the center of the grid. The membrane was then allowed to dry and blotted with primary antibody anti-Aβ peptide (MOAB-2, MABN254, 1:1000, Millipore) in the same manner as described for immunoblotting. This antibody is highly specific for Aβ peptide, with higher selectivity for the more neurotoxic Aβ_42_ compared to Aβ_40_.

### β-secretase Activity Fluorometric Assay

β-site APP cleavage enzyme1 enzymatic activity in hippocampal tissues was measured by the β-secretase activity fluorometric assay kit (#K360-100, BioVision, Milpitas, CA, USA) according to the instructions. The lyophilized active β-secretase was reconstituted by adding 10 μl of ddH_2_O and refrozen immediately at -80°C. 35 μl of fresh hippocampal homogenate from each sample was mixed with 70 μl ice-cold extraction buffer. It was then incubated on ice for 30 min and centrifuged at 10,000×*g* for 5 min. After the centrifugation, the supernatant was transferred to a new tube and the protein concentration of supernatant measured with BCA Protein Assay-Reducing Agent Compatible (Pierce Biotechnology, Rockford, IL, USA). 50 μl of protein extract was added to each well in a 96-well plate. 2 μl of reconstituted active β-secretase with 50 μl of extraction buffer was used as a positive control, and 2 μl of the β-secretase inhibitor with 50 μl sample served as a negative control. The 50 μl of 2× Reaction Buffer and 2 μl of β-secretase substrate was added to each well. The plate was then gently mixed and incubated in the dark at 37°C for 1 h. When the incubation was complete, the plate was read by a Synergy 2 Multi-Mode Reader (BioTek, Winooski, VT, USA) with Ex. 345 nm and Em. 505 nm. Background readings obtained from the blank control were subtracted from all samples. β-secretase activity was expressed as relative fluorescence units per μg of protein sample (RFU).

## Results

### Age- and Sex-dependent Effects of ApoE4 on Learning and Memory in AD Mice

The findings from our RAWM studies, detailed below, support that ApoE4 contributes to profound deficits in learning and memory seen in young (10 months-old) female ApoE4/3xTg mice, which may be associated with hormonal fluctuation initiated around this age range.

**FIGURE 1:**
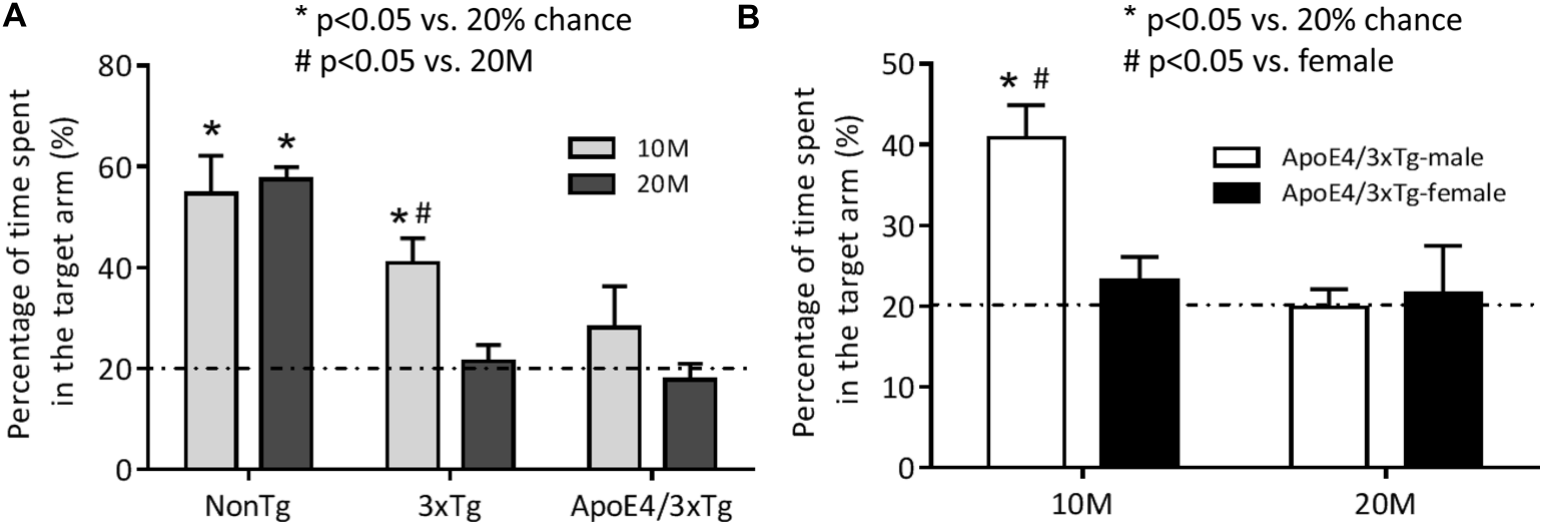
**The spatial memory impairment occurs earlier in female mice carrying ApoE4.** Mice were trained in the RAWM for two consecutive days to learn the location of the target. One hour after the last training, the spatial memory of 10 or 20 months-old nonTg, 3xTg, and ApoE4/3xTg mice was assessed by a 60 s probe trial in RAWM. **(A)** The age-dependent differences in percentage of time spent in the target arm between young (10 month-old) and old (20 month-old) nonTg, 3xTg and ApoE4/3xTg mice. Both 10 and 20 month nonTg mice showed a significant longer time spent in the target arm (^∗^*p* < 0.001) versus 20% chance. In Tg mice, only young 3xTg mice showed a significant difference (^∗^*p* < 0.001) versus 20% chance and a significant difference (#*p* < 0.05) versus old; while young ApoE4/3xTg mice showed a large variation, and neither young nor old Apoe4/3xTg mice showed difference with each other or versus 20% chance. **(B)** The sex-dependent differences in percentage of time spent in the target arm in ApoE4/3xTg mice. When comparing ApoE4/3xTg mice with different age by sexes, only 10 month-old ApoE4/3xTg males demonstrated significant difference with 20% chance (^∗^*p* < 0.001) and with females (#*p* < 0.01). *N* = 8–10 per group. Data is presented as Mean ± SEM. One sample *t*-test (vs. 20%). Two-way ANOVA for age and genotype or age and sex differences.

Probe trial RAWM findings for young (10 months) and old (20 months) nonTg, 3xTg and ApoE4/3xTg mice (**Figure 1A**) showed a significant genotype effect (*F*_2,45_ = 19.27, *p* < 0.0001) and a trend for age effect (*F*_1,45_ = 4.013, *p* = 0.0512). In this “five-arm” maze, young (*p* < 0.001) and old (*p* < 0.001) nonTg mice spent significantly greater than 20% of their time in the target arm. By contrast, only young 3xTg mice spent significantly more time in that arm (*p* < 0.001 vs. 20%; *p* < 0.05 vs. old 3xTg mice). Neither young nor old ApoE4/3xTg mice stayed in the target arm for longer than 20% of the time measured. These data suggest that ApoE4 induces an early learning and memory deficits in AD mice.

To determine sex-dependent ApoE4 effects during aging, male and female ApoE4/3xTg mice in the same age ranges (10 and 20 months) were compared in RAWM (**Figure 1B**). Significant effects of age (*F*_1,33_ = 8.363, *p* < 0.01), sex (*F*_1,33_ = 4.171, *p* < 0.05) and age-sex interaction (*F*_1,33_ = 6.118, *p* < 0.05) on learning and memory were observed. Male, but not female, young ApoE4/3xTg mice spent significantly longer time in the target arm (*p* < 0.001 vs. 20%; *p* < 0.01 vs. female). However, neither old males nor old females exhibited a preference for the target arm.

Among female laboratory rodents of most genotypes, the initiation of irregularity in the estrous cycle occurs at approximately 9–10 months of age (Nelson et al., 1982; Rubin, 2000; Maffucci and Gore, 2006; Brinton, 2012). Using vaginal smear, we assessed estrous stages of our mice as an indicator of their sex hormone changes. In crystal violet staining, three major cell types were observed in vaginal mucosa samples, including nucleated epithelial cells, cornified squamous epithelial cells and leukocytes. The estrous cycle stage was determined by the relative proportion of each cell types seen in the sample (Supplementary Figure S3) (Caligioni, 2009; McLean et al., 2012): Proestrus was characterized by clusters of round nucleated epithelial cells; During estrus, cells were predominantly cornified squamous epithelial cells, occurring in packed clusters; In both metestrus and diestrus, mixed cell types were observed, with great portion of leukocytes and occasionally cornified squamous epithelial cells or nucleated epithelial cells, respectively. Given the smear results, we confirmed in our transgenic animals that cycle length becomes irregular and prolonged (>5 days) as compared to the normal 4–5 days cycle length at around 9–10 months for female mice of all genotypes. Such results are also supported by other studies (Nelson et al., 1982; Maffucci and Gore, 2006)

### ApoE4 Exacerbates Sex-dependent Learning and Memory in Female AD Mice

The effect of ApoE4 in exacerbating hippocampus-dependent cognitive impairment in women has been reported in several clinical studies (Raber et al., 2000; Fleisher et al., 2005; Lehmann et al., 2006). Here, we used RAWM and NAD tests to assess the same ApoE4 effect in transgenic mice with triple FAD mutations, who exhibit both amyloid plaque and neurofibrillary tangles at older age (Oddo et al., 2003). Our findings, detailed below, support that female mice are more vulnerable to learning and memory impairment within the FAD genetic environment, and that ApoE4 further exacerbates this vulnerability.

**FIGURE 2:**
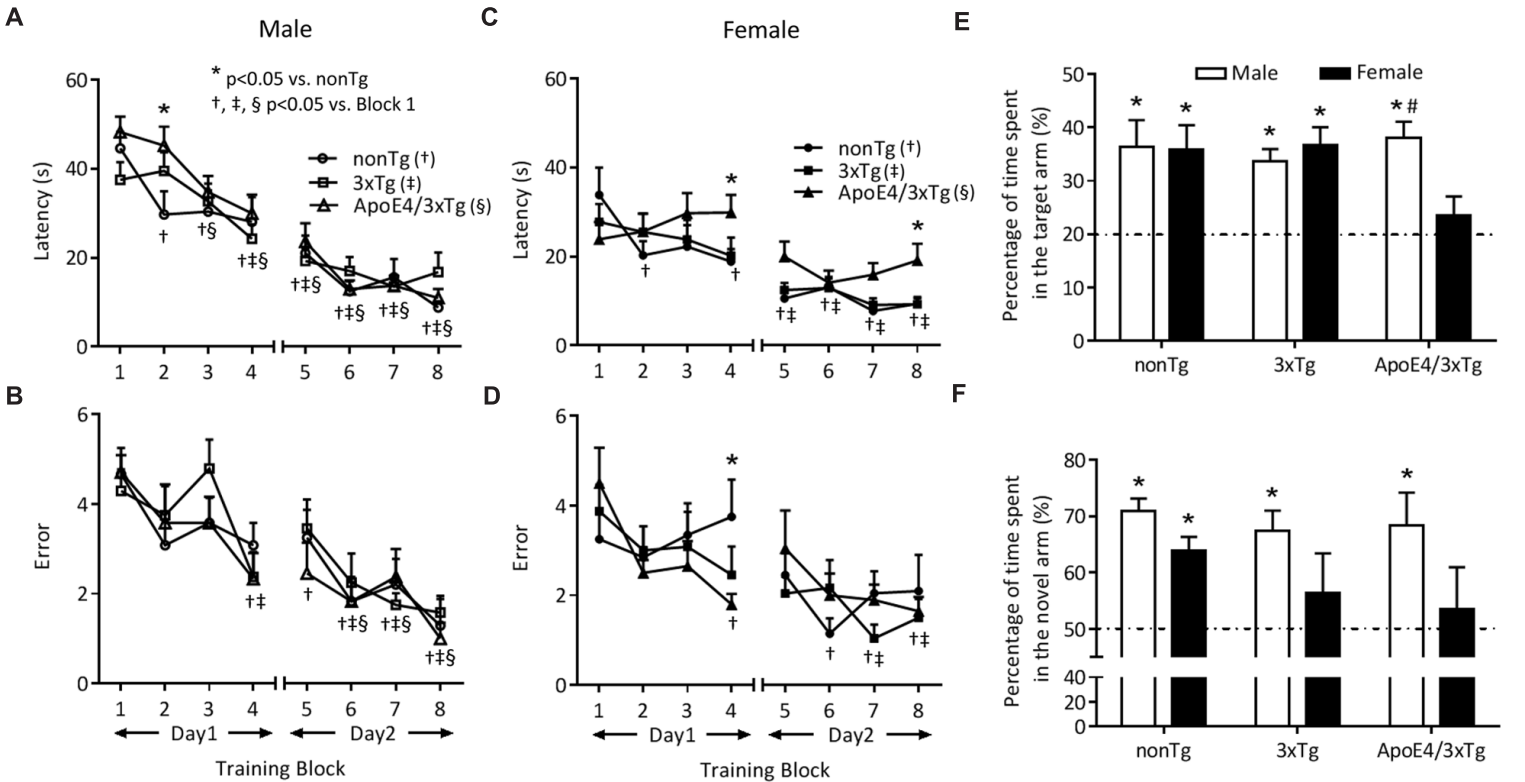
**ApoE4 contributes to the sex-dependent early deficits of spatial learning and memory in RAWM and NAD.** 10 months-old mice were trained for two days (four blocks per day, two trials in each block) in RAWM. **(A,C)** Latency and **(B,D)** errors that mice took to find the target over eight blocks. During the training, male mice of all genotypes showed a significant learning pattern (^†^, ^‡^, ^§^ *p* < 0.05 vs. block1) as indicated by decreased latency **(A)** and errors **(B)**. While in females, the learning pattern was only seen in nonTg and 3xTg mice (^†^, ^‡^*p* < 0.05 vs. block1), but not ApoE4/3xTg mice, as indicated by latency **(C)** and errors **(D)**. ApoE4/3xTg females also showed a genotype difference (^∗^*p* < 0.05 vs. nonTg). One hour after last training, mice were tested in a probe trial. **(E)** Percentage of time mice spent in the target arm during RAWM probe trial. All mice but ApoE4/3xTg females showed a significant difference with 20% chance (^∗^*p* < 0.01), and ApoE4/3xTg mice also demonstrated a sex difference (#*p* < 0.05 vs. female). **(F)** Percentage of time mice spent in the novel arm during NAD retention trial. NonTg mice of both sexes, and 3xTg and ApoE4/3xTg males showed a significant difference with 50% chance (^∗^*p* < 0.05). *N* = 10–12 per group. Data presented as Mean ± SEM. One sample *t*-test (vs. 20 or 50%). Two-way ANOVA for training, genotype and sex differences.

We studied hippocampus-dependent learning and memory in 10-month-old male and female nonTg, 3xTg and ApoE4/3xTg mice using RAWM and NAD tests (**Figure 2**). Two-day RAWM training yielded significant effects on latency (*F*_7,231_ = 40.35, *p* < 0.0001 for male; *F*_7,203_ = 12.9, *p* < 0.0001 for female) and error (*F*_7,231_ = 14.2, *p* < 0.0001 for male; *F*_7,203_ = 5.311, *p* < 0.0001 for female), with significant genotype effect on latency observed only for female mice (*F*_2,29_ = 3.91, *p* < 0.05) (**Figures 2A–D**). **Figures 2A,B** show significantly decreased latency and error over eight training blocks for male nonTg (*p* < 0.05 in block2–block8 vs. block1 for latency; *p* < 0.05 in block4–block8 vs. block1 for error), 3xTg (*p* < 0.05 in block4–block8 vs. block1 for latency; *p* < 0.05 in block4, block6–block8 vs. block1 for error) and ApoE4/3xTg mice (*p* < 0.05 in block3–block8 vs. block1 for latency; *p* < 0.05 in block6–block8 vs. block1 for error), all of which exhibit similar improvements in performance. However, this comparison in females showed improved learning only in nonTg (*p* < 0.05 in block2, block4–block8 vs. block1 for latency; *p* < 0.05 in block4, block6–block8 vs. block1 for error) and 3xTg mice (*p* < 0.05 in block5–block8 vs. block1 for latency; *p* < 0.05 in block7–block8 vs. block1 for error); female ApoE4/3xTg mice did not improve during the training sessions (**Figures 2C,D**). A comparison of each block across all the genotypes studied revealed a significantly longer latency (*p* < 0.05 in block4 and block8) and greater error (*p* < 0.05 in block4) for female ApoE4/3xTg mice as compared to nonTg mice (**Figures 2C,D**). Of note, no difference was observed between nonTg and 3xTg female mice, or between male mice across genotype (**Figures 2A–D**).

In RAWM probe trials (**Figure 2E**), a trend of sex-genotype interaction effect was observed (*F*_2,60_ = 2.853, *p* = 0.0655). All male nonTg, 3xTg and ApoE4/3xTg mice spent significantly more than 20% of the time (*p* < 0.01 for all three genotypes) in the target arm. For females, however, only nonTg and 3xTg mice remained in the target arm for significantly more than 20% of the time (*p* < 0.01 and *p* < 0.001, respectively) (**Figure 2E**). Moreover, when comparing ApoE4/3xTg mice by sex, males lingered for a significantly longer time in the target arm (*p* < 0.05) than did females (**Figure 2E**). No such sex-dependent difference was observed for nonTg or 3xTg mice. However, for 3xTg mice, a difference was noticed when the testing ITI was prolonged to 1.5 h in the Morris Water Maze (Clinton et al., 2007).

We then tested hippocampus-dependent spatial memory deficits by using the NAD test, a behavior test that poses less stress than the RAWM (**Figure 2F**). Our pilot studies showed that a 1 h ITI between the acquisition and retention trials provides the greatest sensitivity for detecting sex-dependent differences in AD mice. In the retention trials that followed initial exposure within two of the three maze arms, a significant sex effect was observed (*F*_1,52_ = 5.714, *p* < 0.05). All male nonTg, 3xTg and ApoE4/3xTg mice spent significantly more than 50% of their time (*p* < 0.001, *p* < 0.01, and *p* < 0.05, respectively) in the novel arm. By contrast, among females, only nonTg mice spent more than 50% of the time in the novel arm (*p* < 0.001) (**Figure 2F**).

### ApoE4 is Associated with More Prominent AD Pathology in the Hippocampus of Female AD Mice

To assess cerebral deposition of neurotoxic Aβ, an early and critical feature of AD, we measured Aβ species expressions, and protein expressions and enzymatic activities of BACE1, the rate-limiting enzyme for Aβ production. Our findings with dot blot, western blot, and β-secretase activity fluorometric assays, detailed below, suggest a sex-dependent ApoE4 effect on increasing BACE1 and Aβ protein expressions, as well as a sex-independent ApoE4 effect on elevating BACE1 enzymatic activities in the hippocampus of female ApoE4/3xTg mice.

**FIGURE 3:**
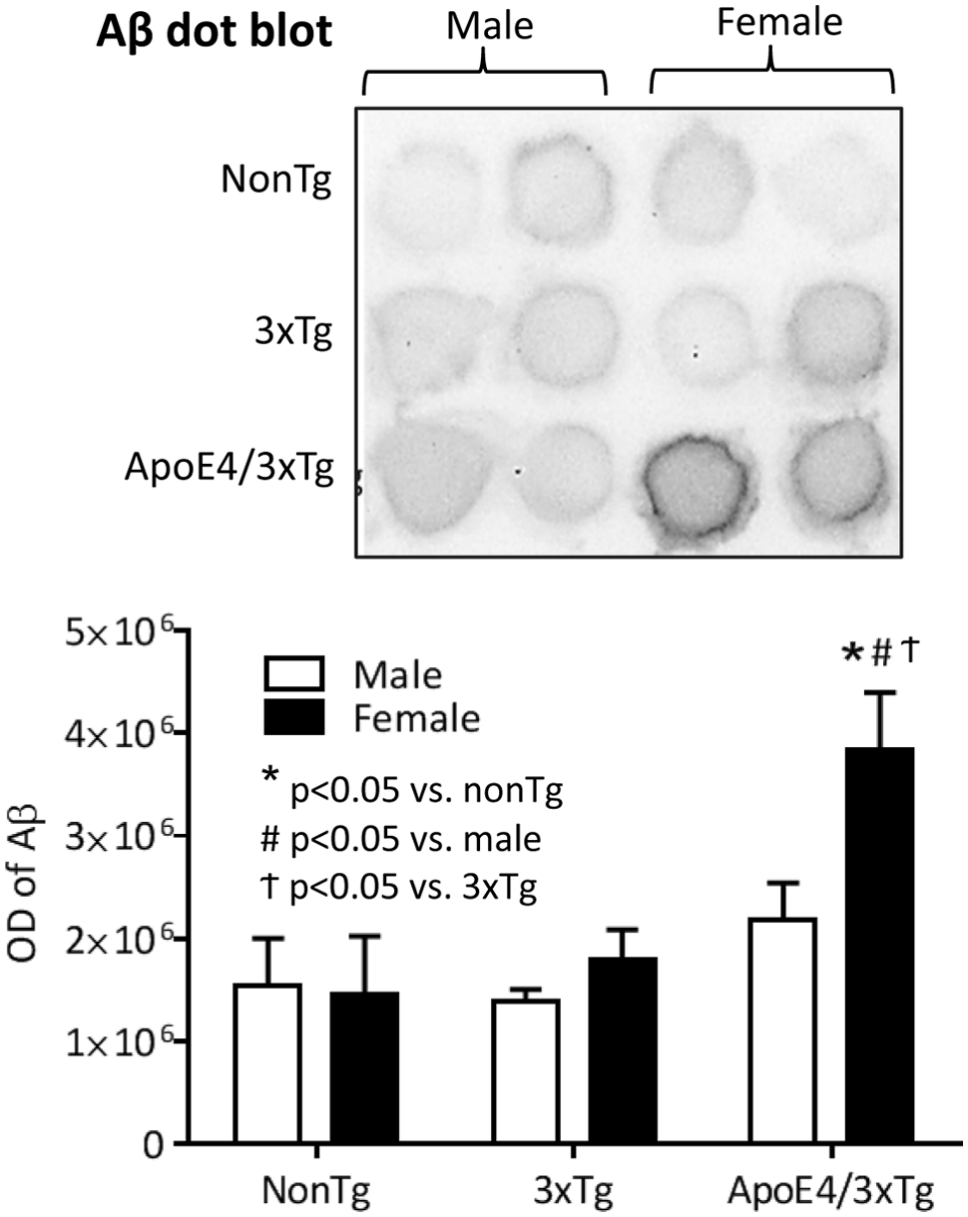
**Female ApoE4/3xTg mice showed more prominent AD pathology in the hippocampus.** Representative image of dot plot on Aβ levels in hippocampus of nonTg, 3xTg and ApoE4/3xTg mice **(upper)**. The optical density of dot plot was presented in the graph **(lower)**. Female ApoE4/3xTg mice demonstrated a significant increase of Aβ (^∗^*p* < 0.001 vs. nonTg, #*p* < 0.01 vs. 3xTg, ^†^*p* < 0.05 vs. male). *N* = 4 per group. Data is presented as Mean ± SEM. Two-way ANOVA for genotype and sex differences.

In dot blot, a significant genotype effect (*F*_2,18_ = 10.68, *p* < 0.001) and sex effect (*F*_1,18_ = 4.588, *p* < 0.05) on Aβ protein expression were observed (**Figure 3**). Male nonTg, 3xTg and 4xTg mice have comparable low Aβ protein expression in the hippocampus. By contrast, female ApoE4/3xTg mice had significantly higher levels of Aβ than both nonTg (*p* < 0.001) and 3xTg mice (*p* < 0.01), as well as significantly higher levels than male ApoE4/3xTg mice (*p* < 0.05).

**FIGURE 4:**
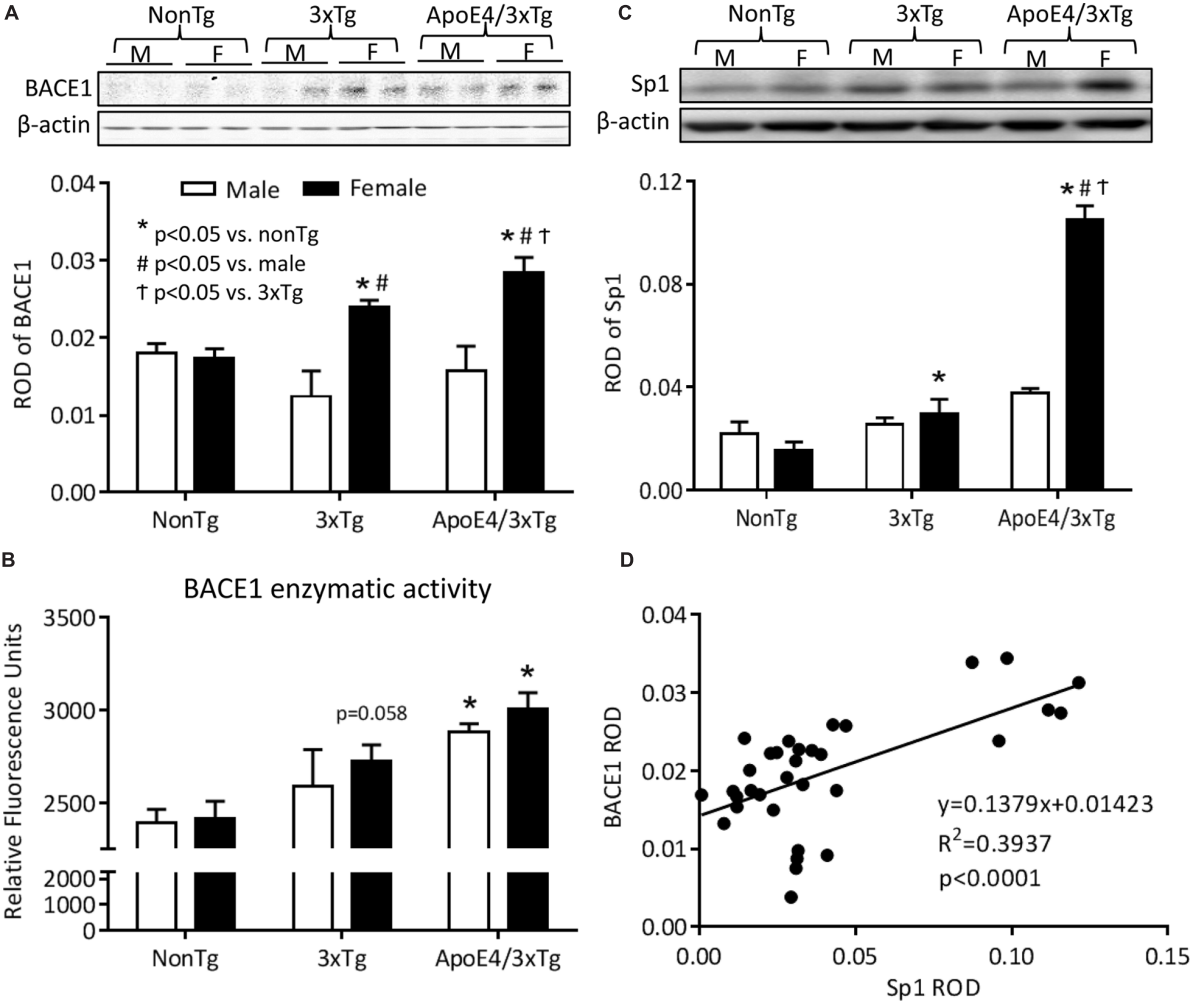
**Female ApoE4/3xTg mice showed elevated BACE1 enzymatic activity and increased BACE1 and Sp1 expression. (A)** The BACE1 enzymatic activity was examined by β-secretase activity fluorometric assay, and was presented as RFU. The BACE1 enzymatic activity was significantly increased in both male and female ApoE4/3xTg mice (^∗^*p* < 0.05 vs. nonTg), while the female 3xTg mice demonstrated a trend of increase (*p* = 0.058). **(B)** BACE1 and **(C)** Sp1 protein expressions were assessed by western blot and was presented as relative optical density (ROD). BACE1 protein levels were significantly increased in female Tg mice, with a greater increase in apoE4/3xTg mice (^∗^*p* < 0.001 vs. nonTg, #*p* < 0.05 vs. 3xTg, ^†^*p* < 0.001 vs. male) **(B)**. The transcription factor Sp1 was highly increased in female ApoE4/3xTg mice (^∗^*p* < 0.001 vs. nonTg, #*p* < 0.001 vs. 3xTg, ^†^*p* < 0.05 vs. male) **(C,D)** The correlation between Sp1 and BACE1 protein expression was examined, and a significant correlation was observed (*R*^2^ = 3937, *p* < 0.0001) in female apoE/3xTg mice. *N* = 3 per group for BACE1 activity assay. *N* = 6 per group for western blot. Data is presented as Mean ± SEM. Two-way ANOVA for genotype and sex differences.

Using β-secretase activity fluorometric assay and western blot, we analyzed BACE1 enzymatic activity and protein expression in mouse hippocampal extracts. In the β-secretase activity fluorometric assay (**Figure 4A**), a significant genotype effect was observed on BACE1 enzymatic activity (*F*_2,12_ = 11.21, *p* < 0.001). BACE1 activity was significantly increased in the hippocampi of both male (*p* < 0.01) and female (*p* < 0.05) ApoE4/3xTg mice. Female 3xTg mice showed a trend of increased BACE1 activity (*p* = 0.058). No sex differences in BACE1 enzymatic activity were observed for any of the genotypes here studied. Findings with western blot (**Figure 4B**) showed significantly effects of sex (*F*_1,30_ = 22.67, *p* < 0.0001) and sex-genotype interaction (*F*_2,30_ = 6.422, *p* < 0.01) on BACE1 protein expression. Male nonTg, 3xTg and ApoE4/3xTg mice had comparable low BACE1 protein levels in the hippocampus; however, both female 3xTg (*p* < 0.05) and ApoE4/3xTg mice (*p* < 0.001) had significantly higher BACE1 expression as compared to nonTg female mice. Moreover, for females, ApoE4/3xTg mice had even higher BACE1 expression than 3xTg mice (*p* < 0.05). When comparing sexes, both female 3xTg (*p* < 0.001) and ApoE4/3xTg (*p* < 0.001) mice had significantly elevated protein levels of BACE1 in the hippocampus than did males of the same genotype.

### ApoE4 is Associated with Increased Sp1, a BACE1 Transcription Factor, in the Hippocampus of Female AD Mice

The BACE1 gene promoter carries several transcription factor-binding sites, one of which is Sp1 which plays a significant role in regulating BACE1 and APP gene expression (Lukiw et al., 1994; Querfurth et al., 1999; Christensen et al., 2004). This increased gene transcription may increase BACE1 protein expression. Using western blot to detect Sp1 expression in the nuclear protein extracts from hippocampus, we found that female AD mice express significantly higher levels of Sp1 protein, with ApoE4 further increasing this elevation in female ApoE4/3xTg mice. The Sp1 expression was also correlated with the BACE1 expression (**Figures 4C,D**).

The western blot result from Sp1 expression shows significant effects of sex (*F*_1,30_ = 42.09, *p* < 0.001), genotype (*F*_2,30_ = 96.86, *p* < 0.0001) and sex-genotype interaction (*F*_2,30_ = 50.96, *p* < 0.0001). Male nonTg, 3xTg and ApoE4/3xTg mice showed no difference in Sp1 expression. However, for females, a higher level of Sp1 was observed for 3xTg mice (*p* < 0.05) than for nonTg mice. Apoe4/3xTg females exhibited a further dramatic elevation of Sp1 expression as compared to female nonTg (*p* < 0.001) and 3xTg (*p* < 0.001) mice as well as to male ApoE4/3xTg mice (*p* < 0.001) (**Figure 4C**). Moreover, we also observed a significant correlation between hippocampal Sp1 and BACE1 protein expressions (*R*^2^ = 0.3937, *p* < 0.0001), suggesting that, as a transcription factor of BACE1, the elevated Sp1 expression may be a molecular mechanism for the increased BACE1 level in female mice in the current study (**Figure 4D**).

## Discussion

To our knowledge, this study is the first to assess age- and sex-dependent ApoE4 effects on learning and memory and AD pathology in FAD mutation background (ApoE4/3xTg mice). Our findings indicate that a sex-linked interaction with ApoE4 may occur across the progression of AD, and that ApoE4 confers greater AD vulnerability in females than in males. The exacerbating role of this allele in females was cumulatively demonstrated in our studies by the earlier and greater degree of spatial learning and memory impairment observed in ApoE4/3xTg female mice, as well as by the increased Aβ deposition, BACE1 and Sp1 protein expression and the increased BACE1 enzymatic activity in the hippocampus of these female mice. Our data suggest that the sex-dependent ApoE4 effects on BACE1 expression and activity may contribute to the Aβ production and be mediated by Sp1 regulation.

Given the baseline concordance of open field and rotarod tests among genotypes and sexes (data not shown), our most striking behavioral finding was observed in female ApoE4/3xTg mice versus that of 3xTgAD and nonTg females. Across the two-day training, the learning behaviors of female ApoE4/3xTg mice yielded flat curves both for latency and for errors in finding the target. In the RAWM probe trials and the NAD retention trials, these females also failed to locate the target arm or the novel arm. These findings suggest a diminished learning and memory function in the context of sex-dependent ApoE4 interactions (**Figure 2**). During the training trials, female mice in general tend to have a shorter latency, especially for the first two blocks, than males (**Figures 2A,C**); given their motor performance, this likely reflected the faster swimming speed measured for these females (data not shown), possibly due to the lower body mass-related higher swimming endurance in females (Dohm et al., 1996).

Parallel to our behavioral findings, we observed a significant increase in the hippocampal Aβ deposition in 10 month-old female ApoE4/3xTg mice, as compared to age- and sex-matched nonTg and 3xTg mice (**Figure 3**). As the critical enzyme for Aβ production, two aspects of BACE1 that tightly associated with its function were examined in our study – protein expression and enzymatic activity (**Figures 4A,B**). Elevated BACE1 protein expressions were found in both female 3xTg and ApoE4/3xTg mice; moreover, a greater increase was seen in female ApoE4/3xTg versus 3xTg mice. This finding raises the possibility that female sex is a major contributor to the upregulated BACE1 expression in 10 month-old AD mice, and the sex-dependent ApoE4 effect yields a further increase of BACE1 expression in females. Moreover, as compared to 3xTg and nonTg mice, both male and female ApoE4/3xTg mice showed a significant increase in BACE1 enzymatic activities, suggesting a sex-independent ApoE4 effect in AD mice. The elevated BACE1 protein expression and enzymatic activity in female ApoE4/3xTg mice may lead to the increased Aβ species and learning and memory impairments observed in this study.

The abnormal regulation of gene transcription implicated in AD pathogenesis (Beyreuther et al., 1992) has been associated with abnormal APP processing, leading to the Aβ increases seen in AD. The increased BACE1 expression we observed may also result from the abnormal gene transcription in AD, with females with ApoE4 gene particularly vulnerable. In our study, sex-dependent differences in transgenic mice were seen at age of 10 months, when irregular cycles begin in female mice (Brinton, 2012), possibly leading to the diminished estrogen signaling and contributing to increased BACE1 protein expression seen in females of both the 3xTg and ApoE4/3xTg strains. Previous studies have shown that ovarian hormones, such as estradiol (E2), can regulate transcription of BACE1 and other secretase enzymes involved in APP processing (Nord et al., 2010). In several transgenic mouse models, E2 has been shown to actively inhibit the BACE1-dependent amyloidogenic pathway (Amtul et al., 2010; Nord et al., 2010; Bernstein et al., 2011), with E2 deficiency resulting in increased BACE1 levels (Yue et al., 2005). A recent study suggests that this E2 action may be regulated through ERs, as ERs mediate luteolin inhibitory effect on BACE1 transcription (Zheng et al., 2015). A further increased BACE1 protein expression in ApoE4/3xTg females may be related to ApoE4 domain interaction induced-endoplasmic reticulum stress (Zhong et al., 2009). The endoplasmic reticulum stress is known to activate the PERK-dependent elF2α phosphorylation, which promotes BACE1 synthesis (Rozpedek et al., 2015).

Transcription factor Sp1 has been demonstrated *in vitro* and *in vivo* to closely regulate BACE1 at the transcription level and to affect APP processing for the generation of Aβ through the regulation of BACE1 protein production (Christensen et al., 2004). Not only does Sp1 bind to the promotor region of target genes, such as BACE1, to activate the gene transcription, but its function and expression are also regulated by E2 and ApoE. It has been shown that ERs form complex with Sp1, and the ER/Sp1 complex regulates target gene transcriptions (Krishnan et al., 1994; Duan et al., 1998) in an ER subtype-dependent fashion (Saville et al., 2000; Safe, 2001). Moreover, ApoE and E2 regulate transcription factor activities associated with Sp1 expression, such as NF-κB (Perez et al., 1994; Barger and Harmon, 1997; Deshpande et al., 1997; Ghisletti et al., 2005) and CREB (Rohlff et al., 1997; Quesada et al., 2002; Choi et al., 2004; Li et al., 2014; Yong et al., 2014). After examining Sp1 expression in our animals, we found its results to be significantly correlated with BACE1 expression, with female 3xTg mice showing a moderately higher Sp1 protein expression and female ApoE4/3xTg mice a greater increase (**Figures 4C,D**). This data further support that sex-dependent ApoE4 effects may arise through an increase in Sp1-mediated BACE1 protein expression, leading to increased Aβ levels. However, Sp1, one of the first identified eukaryotic transcription factors, has been shown to play an important role in regulating the expression of many other genes (Dynan and Tjian, 1983a,b). Further studies are needed to explore the molecular mechanisms underlying this pathway.

Although we show here an elevated BACE1 enzymatic activity in female ApoE4/3xTg mice, and similar results have been reported in clinical studies (Stockley et al., 2006; Ewers et al., 2008), the pathway by which ApoE4 increases BACE1 activity has yet to be demonstrated. ApoE4 has been associated with increased cholesterol levels in both blood (Corder et al., 1993; Notkola et al., 1998) and CSF, as measured by the metabolite 24S-hydroxycholesterol, in AD patients (Papassotiropoulos et al., 2002; Leoni et al., 2006). A further association between increased cholesterol levels and enhanced BACE1 activity has been shown in *in vitro* and *in vivo* studies (Fassbender et al., 2001; Runz et al., 2002). ApoE4 may thus influence Aβ production by modulating cholesterol levels, leading to their increase and hence increases in BACE1 activity (Puglielli et al., 2003; Lahiri et al., 2004). Another possibility is that ApoE4 may increase BACE1 activity by stimulating APP recycling (Ye et al., 2005) and thus increasing intracellular contact of BACE1 to APP.

Sex differences in cognitive deficits and AD pathology during disease progression have been widely described in both clinical studies (Jorm and Jolley, 1998; Andersen et al., 1999; Corder et al., 2004; Barnes et al., 2005; Rosario et al., 2011) and transgenic mouse models (Wang et al., 2003; Clinton et al., 2007; Carroll et al., 2010; Dubal et al., 2012), including 3xTg mice used in current study. Although our RAWM data did not show, 3xTg mice did exhibit a sex-dependent difference in our NAD test and in the probe trial of Morris water maze with 1.5 and 24 h ITI in another study (Clinton et al., 2007). The same study also showed no sex differences in either soluble or insoluble Aβ_40_ or Aβ_42_ levels in whole brain extracts between male and female 3xTg mice at any age from 2 to 20 months-old, as measured by ELISA (Clinton et al., 2007). Similar results were also seen in our dot blot study using hippocampal tissues of 10 month-old 3xTg mice. However, a sex-difference of Aβ load in hippocampal CA1 region appeared in older (12–14 month) 3xTg mice (Carroll et al., 2010). Similar Aβ levels between nonTg and 3xTg mice and increased Aβ levels in female ApoE4/3xTg mice from our results seem to contradict data from others (Oddo et al., 2003, 2009). This discrepancy may be due to using animals with diverse age range, various antibody sensitivity and specificity as well as different experimental methods. ApoE4/3xTg mice used in current study were generated by replacing the endogenous mouse ApoE gene with the human ApoE4 allele in the 3xTg mice (Oddo et al., 2009). By comparing these two mouse strains, we studied the effect of human ApoE4 in murine system with human FAD mutations on learning and memory, BACE1 expression and activity. Further study with 3xTg mice carrying human ApoE2, 3 or 4 allele will be beneficial to distinguish the roles of different ApoE isoforms in Aβ and tau depositions in AD mice brains.

## Conclusion

These data suggest that ApoE4 allele is associated with increased BACE1 enzymatic activity, while female sex plays an important role in increasing BACE1 expression. The combination of both provides a molecular basis for high Aβ pathology and the resultant hippocampus-dependent learning and memory deficits in female ApoE4 carriers. Future studies with ovarian hormone manipulation in ApoE4 female mice are now warranted to probe the mechanisms underlying AD pathology in postmenopausal female ApoE4 carriers, and to develop potential therapeutic targets for this population.

## Author Contributions

Study design; XH, JW, Data Collection; XH, SA, BB, BZ, QZ, MB, Data Interpretation; XH, SA, JW, Drafting of Manuscript; XH, JW.

## Conflict of Interest Statement

The authors declare that the research was conducted in the absence of any commercial or financial relationships that could be construed as a potential conflict of interest.

## Abbreviations

AD: Alzheimer’s disease
ApoE: apolipoprotein E
APP: amyloid precursor protein
Aβ: amyloid beta
BACE1: β-site APP cleavage enzyme
ER: estrogen receptor
FAD: familial Alzheimer’s disease
ITI: inter-trial interval
NAD: novel arm discrimination
PERK: protein kinase RNA-like endoplasmic reticulum kinase
RAWM: radial arm water maze
