# Supplementary material for: Differential contributions of ApoE4 and female sex to BACE1 activity and expression mediate Aβ deposition and learning and memory in mouse models of Alzheimer’s disease
Source: Front Aging Neurosci. 2015 Oct 31;7:207. doi: 10.3389/fnagi.2015.00207 (PMC4628114; doi:10.3389/fnagi.2015.00207)
Supplement: Supplementary file 1 [file Data_Sheet_1.PDF]

Supplementary Material

Differential contributions of ApoE4 and female sex to BACE1 activity and expression mediate Aβ deposition and learning and memory in mouse models of Alzheimer's disease

Xu Hou2, Samuel Adeosun1, Qinli Zhang1, Brett Barlow1, Melissa Brents1, Baoying Zheng1, and Junming Wang1,2,3,4*

*** Correspondence:** Junming Wang: JWang@umc.edu

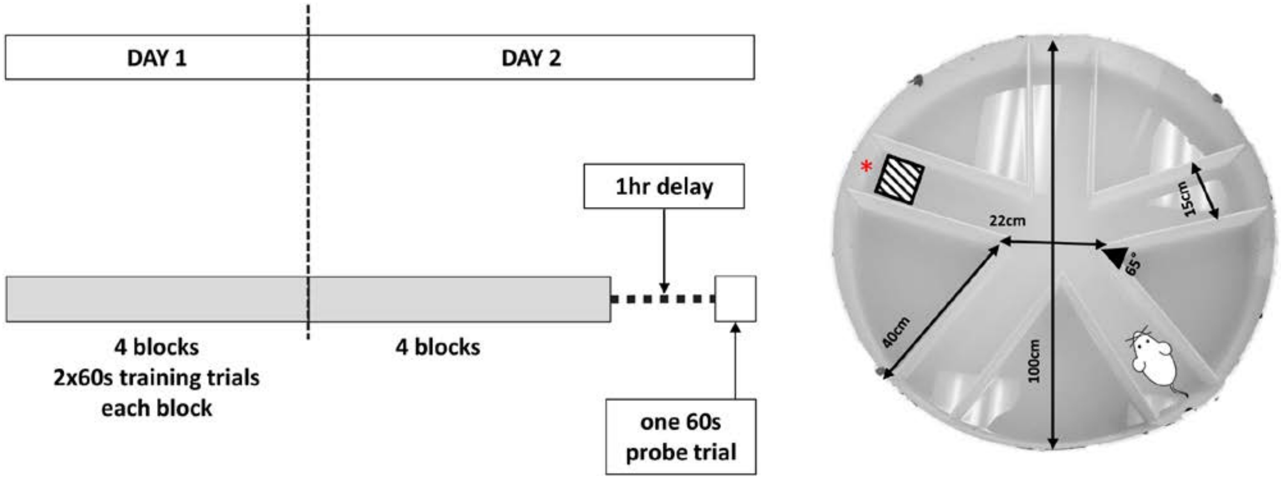

Supplementary Figure 1. The schematic of the radial arm water maze procedure and set up. The Radial Arm Water Maze (RAWM) is used for evaluating learning and spatial/working memory. The apparatus consisted of a 100cm diameter pool, separated into five "arms", each a navigable alley of 40cm length and 15-20cm width leading to a 22cm diameter circle at the pool's center. In one arm (the target arm labeled with *), a 10cmx10cm square transparent platform is placed 5cm from the wall and 0.5cm beneath the water's surface to serve as the target throughout all training trials. During the two days of training, a total of 4 training blocks per day is performed, with two training trials per animal per block. Each mouse is gently placed into one of the four arms (but *not* the target arm) and allowed a maximum time of 60s in which to find the platform hidden in the target arm. One hour after the end of the training, the hidden platform was removed and a 60s probe trial was performed.

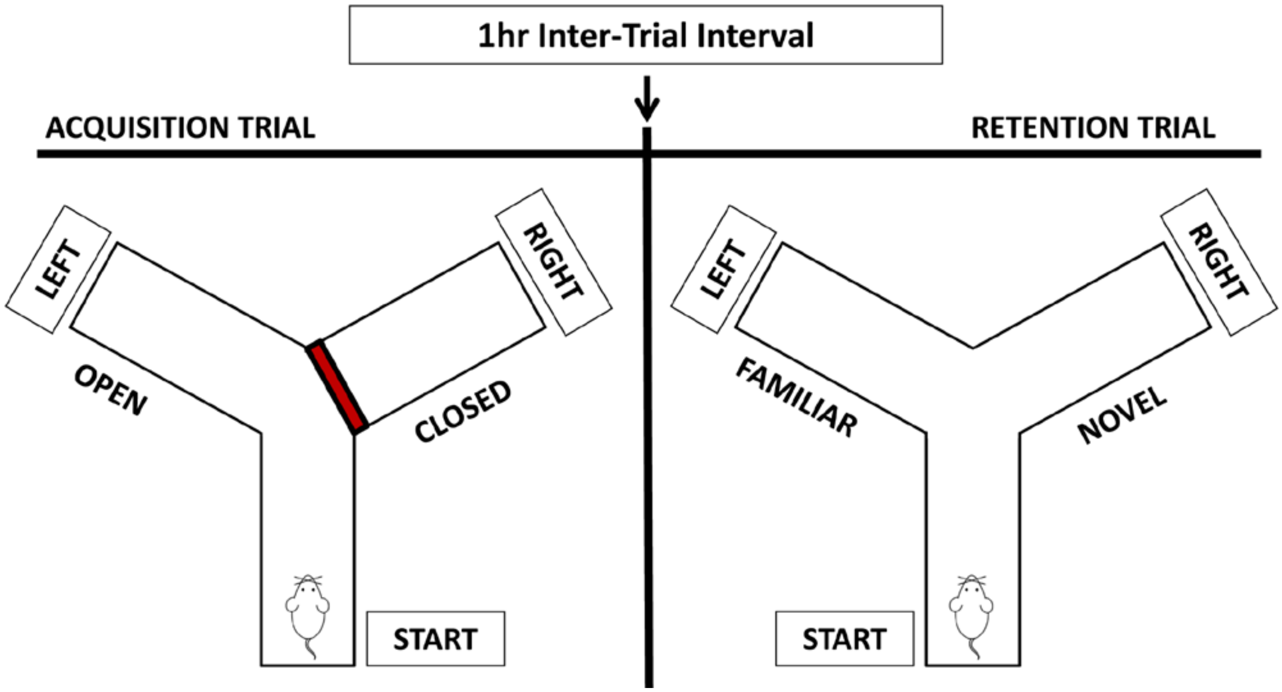

Supplementary Figure 2. The schematic of novel arm discrimination test. The Novel Arm Discrimination (NAD) task is used to study hippocampal-dependent spatial memory. The test is carried out in the Y-maze apparatus, using a floor covered with the wood chippings used in mice cages. Different objects are placed to serve as extra-maze cues at various distances around the maze. The test involved two trials: an acquisition and a retention trial, separated by a 1hr inter-trial interval. During acquisition trials, one arm is blocked (labeled as red). The blocked arm is the Novel arm (N) and the other arm is the Familiar arm (F); the remaining arm is the Start arm (S), which remains open and is kept constant in both trials. In the acquisition trial, each animal is placed in the Start arm of the maze and then allowed to explore both the Start and the Familiar arms (F) for 5min. After 1 h, in the retention trial, the Novel arm (N) is opened and the animal is allowed to explore again for 5min. The walls of the maze are cleaned with 70% alcohol and the wood chippings covering the floor of the maze are mixed before the next animal is placed in the maze. Apart from the Start arm (S), which is kept constant for all animals, use of the Novel and the Familiars arm is counterbalanced among the animals.

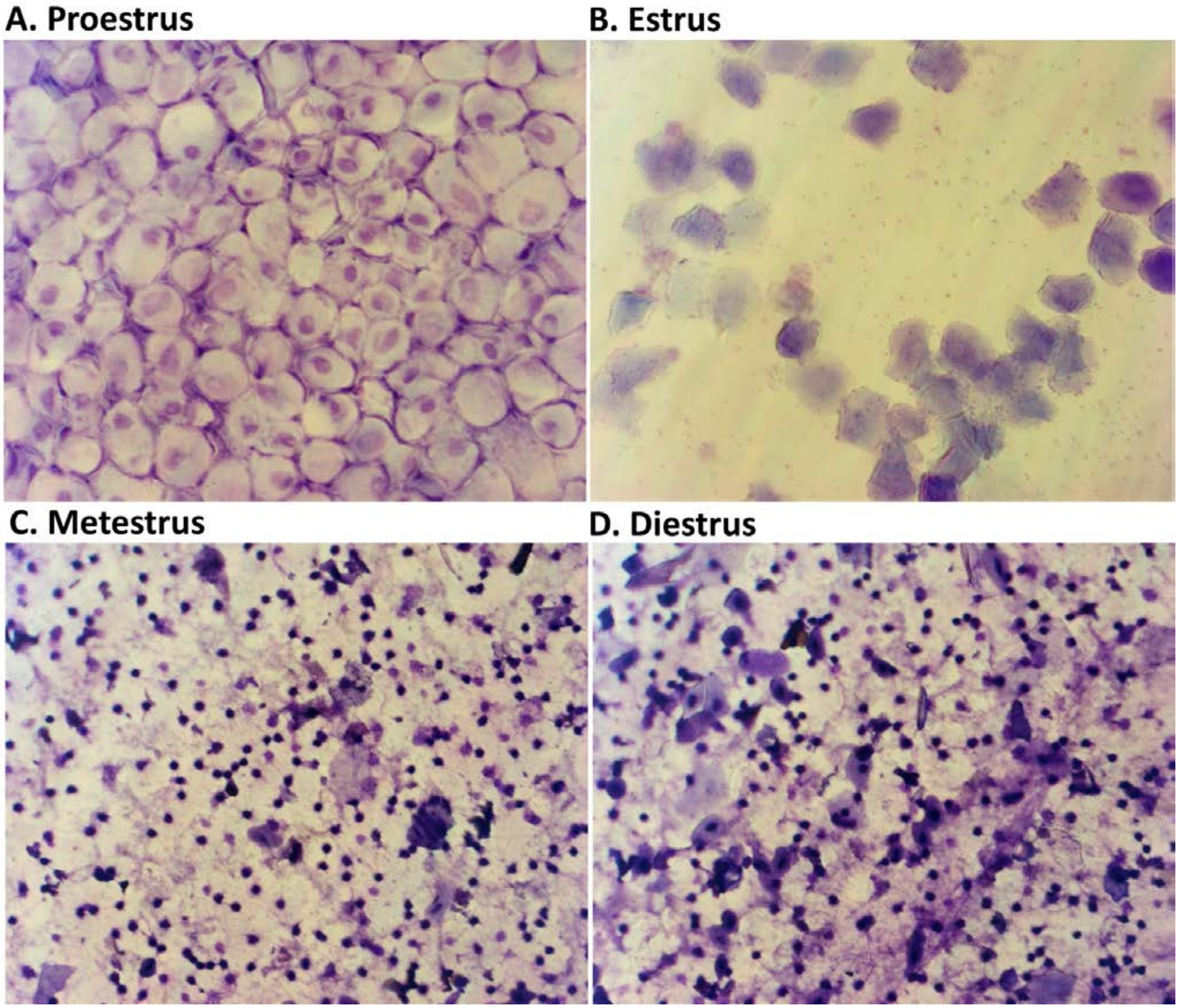

Supplementary Figure 3. Cytological assessment of vaginal smears is used to identify estrous stage. Vaginal mucosa samples were collected from female mice of all three strains (nonTg, 3xTg and ApoE4/3xTg mice) at 10 and 20 months of age. Each sample was then subjected to crystal violet staining and observed under light microscopy. **(A)** In proestrus, cells are predominantly round nucleated epithelial cells, which usually occur in clusters. **(B)** In estrus, cells are mostly cornified squamous epithelial cells, which are also present in clusters. **(C)** In metestrus, mixed cell types are observed with predominantly darkly stained leukocytes and occasionally cornified squamous epithelial cells. **(D)** In diestrus, leukocytes are still predominate cell type; however, a few nucleated epithelial cells can also be present. According to the analysis of estrous stages, majority nonTg, 3xTg and ApoE4/3xTg mice at 10 and 20 months of age have irregular and prolonged cycle length as compared to the normal 4-5 days cycle length seen in young mice.
